# Beliefs about hypertension among Nigerian immigrants to the United Kingdom: A qualitative study

**DOI:** 10.1371/journal.pone.0181909

**Published:** 2017-07-27

**Authors:** James Tosin Akinlua, Richard Meakin, Nick Freemantle

**Affiliations:** Department of Primary Care and Population Health, University College London Medical School, London, United Kingdom; Western Sydney University, AUSTRALIA

## Abstract

**Objective:**

The aim of the study was to elicit beliefs about hypertension among Nigerian immigrants in the United Kingdom.

**Background:**

The distributions of cardiovascular risk factors and diseases are not shared equally across ethnic and economic groups in the United Kingdom. Its burden is more clustered among minority ethnic populations and migrant groups including black African Nigerian migrants. Similar patterns have been reported across Europe, Australia, Canada, Nordic countries and the United States of America. There are about 300 distinct ethnic groups in Nigeria and reliable information about their beliefs about hypertension is not available. Given that the United Kingdom has a large community of Nigerian immigrants from these different ethno-cultural backgrounds, understanding their unique beliefs about hypertension may help promote appropriate care for this population in the United Kingdom and Nigeria.

**Setting:**

A single Pentecostal church community in West London

**Participants:**

Twenty-seven Nigerian migrant members of the church entered and completed the study

**Methods and outcome measure:**

A qualitative interview study was conducted. The interviews were analysed using thematic framework analysis. The outcome measures were emerging themes from the thematic framework analysis.

**Results:**

Participants expressed beliefs in four major areas related to hypertension: (1) The Meaning of the term hypertension, (2) Perceptions of causation, (3) Effects of hypertension, and (4) Perceptions of treatment. The study revealed a diversity of beliefs about hypertension which incorporated both orthodox and culturally framed ideas.

**Conclusions:**

This study identified important beliefs among Nigerian migrants about hypertension that can contribute to our understanding of the management of hypertension in this group and suggests the need for further research to determine whether these beliefs may be representative of this group.

## Introduction

The distributions of cardiovascular risk factors and diseases are not shared equally across ethnic and economic groups in the United Kingdom. Its burden is more clustered among minority ethnic populations and migrant groups including black African migrants [1–4]. Similar patterns have been reported across Europe, Australia, Canada, Nordic countries and the United States of America [5–14].

The explanations for this gradient in the distribution of cardiovascular risk factors are complex and involve interplay of several factors. Studies have shown that the health status of migrants to high income countries may change overtime, sometimes becoming worse than host communities [15]. The change in health status of migrants is dependent on a number of socio-cultural factors including age, gender, migration status, ethnicity, unemployment, stress and poor housing quality [15]. Furthermore, individual life style choices such as diet and health behaviours may be influenced by migration leading to increased prevalence of chronic diseases among migrant populations [15].

According to the recent Health Survey England 2014 report, the prevalence of hypertension in England among all adults who are 16 years and above is 29.6% [16]. This report concurs with previous studies [17–19] that suggest that higher blood pressure is observed among both migrants of African and black African-Caribbean origin compared to other ethnic groups. While rates of hypertension control in England have improved slightly over the years, 51% of people with high blood pressure still remain untreated and only 27% of hypertensive patients on treatment achieve blood pressure goals set by established guidelines [20]. Several studies conducted in the United Kingdom have shown poorer blood pressure control among minority black populations [21–27].

Although, theories of acculturation-that is adoption of norms, behaviours and beliefs prevalent in the receiving society have been used to explain changes in behavioural patterns of migrants that affect their health outcome negatively, linear acculturation may not always explain patterns of migrant health behaviour overtime [28, 29].

It is important to consider the individual who is adopting the health behaviour, barriers to adopting the behaviour as well as pre-migration health status and epidemiological patterns of Non Communicable Diseases in countries of origin [30]. Therefore, understanding how African migrant minority groups construct and interpret hypertension, in order words, their beliefs about hypertension is an important first step that should guide development of interventions geared towards reducing inequality in health outcomes of this group [15, 31–37].

However, studies that have explored health beliefs and health behaviours of minority migrant populations tend to aggregate groups across different countries of origin which may not reflect peculiarities in beliefs and behaviours in different groups [38]. Nigerians form a substantial part of the black minority population in the United Kingdom. However, migrants from Nigeria may belong to one of over 300 distinct ethnic groups. These ethnic groups may have both shared and peculiar beliefs. But, to our knowledge, studies that have specifically explored beliefs about hypertension among Nigerian migrants (known hypertensives and those not known to be hypertensive) in the United Kingdom are very scarce and only one study has described the beliefs of diagnosed hypertensives living in Nigeria. Therefore, the objective of this study is to elicit beliefs about hypertension among Nigerian immigrants in the United Kingdom.

## Methods

The study design was a qualitative interview study examining the beliefs of Nigerian immigrants to the UK living in London. The principal researcher (JA) belongs to the Yoruba ethnic group, one of the ethnic groups in Nigeria, and is a member of the church community where the study was conducted. JA is also a medical doctor. This study was supported by the President’s Nigerian Youth Service Corps scholarship award.

### Ethics

Before commencement of the study, ethics approval was received from the University College London Ethics committee (Project ID number 7811/001). Permission to conduct the study was given by the Pastor of the church. Both written and verbal informed consent was obtained from participants. Interviews were conducted privately and participants’ transcripts were pseudo-anonymised.

### Relationship between researcher, participants and the church

The researcher and participants had a pre-established relationship as they were members of the same church community and some had worked together in the church in areas such as publicity and Sunday school teams. The interviewees were aware that JA is a medical doctor and a PhD student. This could have made the interviewees uncomfortable expressing their beliefs, if they thought that JA would judge whether they had adequate knowledge about hypertension. However, participants were told that there were no right or wrong answers. As JA is a Nigerian and currently a member of the community being studied, it was necessary to consider his relationship with the church. In the context of the study JA was not acting in a medical capacity and any participant who expressed a concern about their blood pressure was given an information leaflet at the end of the interview and advised to seek further advice from their general practitioner.

### Context and sampling

The study was either conducted in the church building or a convenient venue for participants. The study took place over a period of 3 months. JA recruited a convenience sample of 27 participants from the church. The church is located in the West London. More than 95% of the church membership is black and 93% of them are black Nigerian immigrants to the UK. The church consists of Nigerian immigrants from different cultural backgrounds with most of them having post graduate degrees. All members who were approached consented to take part in the research and every eligible participant (i.e. Nigerian members greater than 18 years old) was offered a chance to take part. Selection of participants for interview stopped when data saturation was achieved.

### Data collection

Data were collected using semi-structured interviews. However, JA recorded reflections and comments about the interviews in a diary. All iterative modification to the data collection and analysis were recorded in this diary and participants were notified about the use of a reflective diary.

### Semi-structured interviews

People’s beliefs about illnesses can be understood from different viewpoints but the anthropological perspective may be very helpful when the intent is to elicit and compare cultural understandings. Kleinman’s anthropological explanatory models (EM) of diseases categorized people’s beliefs about illness into the cause of an illness, its course, diagnosis, symptoms and treatment [39, 40]. Marked differences between Health Care Provider (HCP) and patients’ EMs about hypertension have been identified in previous studies [30, 33, 41–44]. Similarly, associations between patients’ EMs about hypertension and adherence to prescribed medicines and life style changes have been well documented [33]–[36].

Therefore, face to face semi-structured interviews with open-ended questions addressing patients’ beliefs about HTN were conducted by JA using a standard interview protocol which composed of eight questions adapted from Kleinman and Weiss’ Explanatory Model Interview Catalogue [39, 40, 45].

All interviews were conducted in English as all church members are proficient in the English Language. The interviews were recorded and lasted up to 45minutes.

### Data analysis

The interviews were transcribed verbatim. The transcribed data and the reflective diary formed the basic data used in the analysis. The data were manually analysed thematically using the framework analysis approach. The analysis was done concurrently with the interviews allowing emerging themes from preceding interviews to inform later interviews. The process of data analysis was as follows:

1. *Familiarization with the raw data*: this took place through manually transcribing all the data and reading the manuscripts, reflective diary and comments from member checking process. This process allowed JA to identify emerging concepts from the data.
2. *Identifying a thematic framework*: JA labelled the transcript with identified index of emerging themes. The transcript was then divided into sections corresponding to well-established themes. This formed the backbone of the thematic framework.
3. *Coding the transcripts according to themes identified*: this next level of analysis involved highlighting transcript with different colour codes representing themes.
4. *Charting the data*: this process was done by cutting and pasting highlighted sections of data into respective thematic headings
5. *Mapping and interpreting the data*: this level of analysis involved examination of the relationships and interaction between themes identified

Initial analysis was done by JA. The data was also independently analysed by RM and similar themes were identified by both authors. A final interpretation was agreed upon following discussion.

### Member checking

After transcription of recorded interviews and analysis of data, participants were sent a copy of the transcript and analysed data for their comments. All participants agreed with the content of the document.

## Results

### Participant characteristics

A total of 27 individuals participated in the study. Two interviews were unfortunately lost due to corrupted audiotapes bringing the final sample to 25 participants. In this sample, only 2 participants were known to be hypertensives while others were not known to be hypertensive. Participant characteristics are described in Table 1.

**Table 1:** Characteristics of participants (N = 27).

	N (%)
**Gender**	
Male	12(44%)
Female	15(56%)
**Age (Median (min, max))in years**	
Men	35(24,73)
Women	30(22,51)
**Ethnicity**	
Yoruba	10(40%)
Igbo	4(12%)
Hausa	3(8%)
Tiv	5(20%)
Urhobo	2(8%)
Okpameri	1(4%)
Ijaw	2(8%)
**Highest education level achieved**	
Secondary	1(4%)
College or Graduate education	9(33%)
Post Graduate degree	17(63%)
**Blood pressure status**	
Known hypertensive	2(7%)
Not Known to be hypertensive	25(93%)

## Beliefs about hypertension

The analysis yielded the following four overarching themes about hypertension as shown in Table 2: (1) Meaning of the term hypertension, (2) Perceptions of causation, (3) Effects of hypertension, and (4) Perceptions of treatment.

**Table 2:** Themes, subthemes and concepts.

Themes	Sub-Themes	Concepts
The meaning of the term “hypertension”	Ethnic Understanding	HTN means high blood volume in my local language(n* = 12)
		HTN means burning or boiling blood in my local language(n = 9)
		HTN means resistance to blood flow in my local language(n = 1)
		HTN means rising blood or blood shooting up in my local language(n = 6)
	Biomedical Understanding	HTN is raised blood pressure as measured by an instrument (n = 9)
		HTN is a severe form of disease while high BP is the mild form (n = 2)
	Hyper–Tension	HTN equals too much stress or thinking too much(n = 11)
Perceptions of causation	Bio-medical Causation	HTN may be caused by life style and old age (n = 21)
	Stress	HTN is caused by high stress levels and stress is caused by thinking too much and not having enough rest (n = 23)
	Hereditary	HTN is a result of genetic inheritance (n = 7)
		HTN is caused by learning wrong behavioural patterns from one’s parents (n = 2)
	Ethnic Models of Causation	HTN is caused by anything that leads to resistance to blood flow (n = 1)
The effects of hypertension	Symptoms of Hypertension	HTN is announced by feelings like intermittent headaches, fast heart beat, dizziness and loss of appetite (n = 20)
		Absence of symptoms indicate absence of HTN (n = 6)
	Illness caused by hypertension	HTN causes unnoticed harm, burst blood vessel resulting in stroke or paralysis, damage to the heart, and sudden death(n = 22)
		HTN causes bad dreams and mentally disordered behaviour (n = 4)
Perceptions of treatment	Cure versus Management	It is not certain if HTN can be cured but it can be managed (n = 6)
	Therapeutic Agents	Use of orthodox medication only is effective for treatment of HTN (n = 5)
		Use of orthodox and herbal medication together is effective for treatment of HTN (n = 8)
		Orthodox medications in addition to life style changes are the best way to treat HTN (n = 20)
		Orthodox medication are not effective and should not be trusted(n = 2)
	Dealing with the underlying cause	Apart from taking orthodox and herbal remedies, underlying causes like stress needs to be addressed to treat HTN effectively(n = 4)
	Spiritual Help	HTN can be cured if you are spiritually minded (n = 3)

*n refers to the number of respondents whose perceptions contribute to the corresponding concepts.

### The meaning of the term “hypertension”

There were varieties of beliefs about the meaning of the term “hypertension” among participants. This theme was divided into 3 sub-themes; Ethnic Understandings, Biomedical Understandings and Hyper-Tension.

There was overlap between beliefs among participants. Eleven participants held both ethnic and biomedical understandings of hypertension. One participant (P4) expressed both biomedical understanding and belief that hypertension meant “Hyper- Tension”. Both diagnosed hypertensive participants (P9 & P10) shared all the three understandings about hypertension.

However, the two participants who had been diagnosed by their doctor believed that they were not hypertensive because they believed that it was a state of mind and therefore adjusting one’s state of mind removed the diagnosis. Since these two participants held all the three understandings of hypertension this may be congruent with holding a belief that hypertension was “hyper-tension”.

“Well I am said to have it by doctors but personally I feel it’s the condition of one’s mind and so in my mind I resist it”

*(p10; 004)*.

“Well recently when I went for check up they said I had hypertension… but I don't believe I have”

(p9; 010–014)

#### Ethnic understandings

Although each study participant belonged to one of 7 ethnic groups, only 4 ethnic understandings of hypertension were identified namely; ‘Raised or high blood volume’, ‘Burning or boiling blood’, ‘Resistance to blood flow’, and ‘Rising blood or blood that shoots up’. Mostly, these meanings were translations of the local ethnic names for hypertension or high blood pressure. In all interpretations of local ethnic languages for hypertension, reference is made to the blood as the affected entity in the body; but they differ slightly in their explanation of the underlying abnormality.

The concept of raised or high blood volume being the meaning of hypertension was shared by about half of the participants particularly among those from Yoruba and Tiv ethnic backgrounds.

“*Ahhh…in my own language we call it eje riru… I think the volume within the blood vessels is raised yeah”*

(p1; 011–015)

*“Ehm*…*we call it "awambe-atave"*.*It means the blood is high… Well this refers to the volume of blood being high or raised”*

(p9;018–022)

The explanation of burning or boiling blood was peculiar to the majority of Yoruba ethnic group participants

“…Yeah we call it eje-riru meaning …basically when your blood is just burning or boiling”

(P3; 013)

The notion that hypertension meant resistance to blood flow was expressed by only one participant, the participant being one of the two known hypertensive participants of this study. This notion is a different interpretation of the same local name for hypertension among the Tiv ethnic group.

*“It is some form of resistance in blood flow in the veins*, *that is*, *something that I think it should be*. *In my language it’s called “awambe-ataver… Well I think it is some form of narrowing in the veins which leads to obstruction to the flow of blood due to anything*. *Don’t know what causes the narrowing but I think this is the situation*. *Well that’s the way I look at it.”*

(P10; 008–011)

There was overlap in ethnic understandings about hypertension as some Yoruba and Tiv ethnic participants gave similar meanings to different local names for hypertension as well as different meanings to similar local names.

Rising blood or blood that shoots up’ was the meaning given to hypertension by participants from Hausa and Igbo ethnic groups respectively.

*“It is called "hawan jini" in hausa*. *It means…high*..*ehm…hawan-thats you're rising*, *jini-is blood so rising blood yeah”*

(p22;039–040)

*“Yeah we have a name in my dialect*. *Obara-means blood*, *mgbanyi-enu-this means to shoot up so blood that shoot up”*

(p23;022)

#### Bio-medical understanding

Some participants expressed a biomedical understanding of the term hypertension in that they believed hypertension was raised blood pressure as measured by an instrument usually by a health professional.

“Yes that's all I know about it… by measuring it and pressure is high”

(p4; 012)

However, two participants who expressed biomedical understandings believed that hypertension was a separate construct from high blood pressure. In essence, they believed high blood pressure referred to the mild form of the ailment while hypertension was a severe form of the disease.

“*If it is just like ehm… one figure above the normal that's is high blood pressure when…It is when it goes up higher and there's an alarm*, *it leads to hypertension and then to stroke”*

*(p25;072)*.

#### Hyper-tension

Some participants simply said hypertension means “Hyper–Tension” (i.e. hypertension equals too much stress or thinking too much)

*“…Ehm*, *high blood pressure is high stress level”*

(p5; 041)

### Perceptions of causation

Participants expressed several beliefs about causes of hypertension. These were grouped into 4 sub-themes; “biomedical causation”, “stress”, “hereditary” and “ethnic models of causation”. Most participants held more than one belief about the causation of hypertension. In particular, most participants shared both biomedical and stress related causes for hypertension.

#### Bio-medical causation

Biomedical causes refer to known risk factors for hypertension that have been identified by medical science. These include life style choices such as diet, physical activity, excessive alcohol intake, smoking and being overweight and other non-modifiable risk factor like old age. Most of the participants believed that all these risk factors could be a cause of hypertension.

*“Ahm… In the first place there are certain things that can give rise to hypertension*. *One*, *we believe that if somebody is heavily built*, *very fat people*, *you know they are prone to hypertension”*

(p1;021–022)

“…Junk food then too much of salt intake…”

(p2; 020)

Some participants believed that certain life style habits such as taking too much caffeine and taking recreational drugs were causes of hypertension.

"…I think caffeine as well…”

(P6; 053)

"I think taking drugs like cocaine…”

(P2;018)

Some participants thought that it was a natural consequence of the aging process.

*“From my own personal experience*, *I don’t know much… I don’t know much about blood pressure*. *One is because another answer to my culture is that they believe it is older people that have it… like middle age people”*

(p4; 033–036)

#### Stress

Most of the participants believed that high stress levels were a cause of hypertension and that the stress could be caused by thinking too much, working too much without taking a rest, targets not being met, being depressed as a result of losing a loved one, lack of money and worrying about a various issues of life.

“I think the major thing is stress and stress come in through worries

(p5;050)

Some participants expressed the view that stress may not cause permanent raise in blood pressure. Blood pressure may go back to normal when stress level reduces.

*“Yes I would classify worry under stress*. *But*, *I wouldn’t say it’s a constant thing*. *Like your pressure might be high at a particular period like if you are going thru a loss*, *your pressure might be high then*, *and it might only be high for the first couple of days after when you started accepting*, *maybe when you are in denial it might be high then it might go down”*

(p4;042–044)

One participant believed that certain foods cause pressure on physical nature leading to emotional stress but that different foods affect people differently.

*“I think there are some foods that may put pressure on you and the physical nature will translate to emotional stress as well*. *I would not be particular about the food but I think it will be specific to a person… ”*

(P8; 046–051)

#### Hereditary

A number of participants believed that the cause of hypertension could simply be genetic. In other words, the blood pressure genes get passed down from parents to siblings.

“I think it can be passed down from parents…”

(P4; 108)

Two participants thought that hypertension was not necessarily genetically inherited but that you learned certain behavioural patterns such as the way you handled stress from your parents.

*“…Like basically not everyone will be born to deal with situations in the most appropriate way*. *Some people are just born to be stressed*, *so I wouldn’t say that is genetics*, *if your parents can not handle stress*, *you might take that from them and not be able to handle stress as well.”*

(P3; 017–019)

*“Well I wouldn't say it’s directly it is because of the genes I would say it’s because you take traits from your parents*. *So the way they react or act to certain things might affect you… It’s not like there's hypertension in this person’s blood and it is passing*. *It’s more like behaving in a certain way that is similar”*

(P8; 122–129)

#### Ethnic models of causation

One of the diagnosed hypertensive participants expressed what his community believed was the cause of hypertension. He thought that it was due to anything that causes resistance in blood flow.

*“We believe that anything that could cause clotting of blood in your vein could cause the slowness of flow of blood… Maybe eating a lot of fat may be harmful*, *smoking may be harmful*. *Each of these things could reduce flow of blood causing hypertension”*

(P10; 013–018)

There seemed to be a direct relationship between beliefs about meaning and the cause of hypertension. This is evident among those who believe hypertension means high stress level and participant (P10) who believed hypertension meant resistance to blood flow.

### The effects of hypertension

Participants’ beliefs about effects of hypertension are divided into 2 sub-themes: “Symptoms” and “Illness caused by hypertension”.

#### Symptoms

Symptoms referred to certain feelings in the body that indicated the presence of HTN.

Many participants including those who had been diagnosed with hypertension asserted that intermittent headaches, fast heart beat, dizziness and loss of appetite are signs of raised blood pressure. Many participants who held these beliefs about the symptoms of hypertension also held stress related beliefs of causation.

“Yes severe headache is a big sign of hypertension”

(p24; 091)

*“When stressed*, *the heart starts beating fast…”*

(p16; 093)

However, some participants thought that absence of symptoms indicate absence of hypertension.

“What else can i say…ehmm…cause i look healthy and feel well so i don’t think i have it so…

(p2;007)

#### Illness caused by hypertension

Many of the participants described complications that have been identified by medical science such as unnoticed harm, burst blood vessel resulting in stroke or paralysis, damage to the heart, and sudden death.

*“What I fear most about it is the fact that it doesn’t give a moment notice before it strikes and sometimes some people don’t know they have it*. *They will be going about their daily activities until they are completely down*. *That is when they would go to the hospital and they will now find out”*

(p23; 066–067)

“I think it can lead to stroke…”

(P2; 035)

“The main thing is may be stress on your heart…”

(P4; 073–074)

However, some participants expressed non-orthodox ideas about illnesses that could be caused by hypertension such as bad dreams and mentally disordered behaviour.

“… It may cause bad dreams”

(p15; 060)

“T*o me hypertension can still lead to having mental disorder*. *Like you being lunatic*. *That is having a lunatic display“*

(p17; 120)

### Perceptions of treatment

Participants’ perceptions of treatment for hypertension were grouped into 4 sub-themes;

“Cure v Management”, “Therapeutic Agents”, “Dealing with the underlying cause” and “Spiritual Help”

#### Cure v Management

While many believed that hypertension could be cured by using drugs, making life style changes or seeking spiritual help, some participants believed that hypertension could only be managed and not cured. The beliefs of those who said hypertension could only be managed seemed to be influenced by the experience of those in their social network who had the disease.

*“Well since I don’t think it is curable to start off with… But actually*, *Grandma has continued to take drugs for years”*

(p11; 069–070)

#### Therapeutic agents

Only a few participants believed that use of orthodox drugs alone was effective. The majority of participants believed that orthodox medications in addition to life style changes are the best way to treat HTN. However, some believed that using orthodox drugs and herbal remedies together were more effective than orthodox medicine alone.

*“Yeah*. *You can get tablet from doctors and you can also use herbal medication as well*. *That is what we use in Africa for most of us”*

*(p12; 100)*.

*“I think the orthodox ones are better but the herbal remedies too can help*. *You can just supplement with it*. *It’s not going to be the major treatment”*

(p15;105)

Two participants (not known to be hypertensive) thought that orthodox drugs were not effective and would not take them no matter what.

*“Well*. *I don’t think orthodox drugs are effective*. *Because if they were effective those of them who go to hospital should have had the illness cured or be gone forever or be gone for a long time*. *they still have to be going and coming so in that case it only subdues it*.*so it is not effective”*

(p18;064–065)

One of these two participants also believed that eating special foods can cure hypertension.

“*Ok*. *I have different ideas with the treatment because I have someone that has hypertension*. *Like celery leaf*, *garlic*, *garlic juice*, *water melon seed*, *ehm…*..*banana*, *eating of banana”*

*(p2; 037–044)*.

#### Dealing with the underlying cause

Some of participants who expressed the view that orthodox and herbal remedies could be used in treating hypertension believed that if underlying factors such as stress and life style habits were not addressed, these treatment modalities would be ineffective.

*“I think the major thing is before you treat an illness or a sickness*, *you must know the cause and it applies to if not all*, *everything when you know the problem then you know where to start from*. *From my own knowledge I believe that hypertension is caused mostly by the stress level in mankind*…*so once those things are known and addressed then systematically or simultaneously you can prescribe drugs and at the same time give the carrier the kind of encouragement they need”*

(p5;129–132)

#### Spiritual help

Some participants believed that hypertension could be cured by spiritual means. There was no specific relationship pattern between those who share this belief and their beliefs about causes of hypertension.

“For me, I am a spiritual person because I believe in Christ, so I believe that Christ also heals. So I believe that with Christ in me, I can be healed of anything”

*(p7; 104)”*.

## Discussion

There have been other studies which investigated the beliefs about hypertension among ethnic minority groups in the United Kingdom [15, 20–27, 46] and other developed nations [37, 43, 47]. There have also been studies which investigated the beliefs regarding other chronic health conditions among immigrant communities in developed countries [48, 49]. However this is the first study that has explored the beliefs, perceptions and attitudes regarding hypertension among Nigerians living in the United Kingdom. It is important to note that an overwhelming majority of the participants in this study were not known hypertensives. Only two participants were diagnosed hypertensive. Therefore the views presented may not be completely representative of those who live with the disease but beliefs of those not known to be hypertensive may be very useful in giving insights into beliefs, attitudes and behaviours regarding hypertension that exist in the community and provide the background against which people who are diagnosed with hypertension develop their understanding of the condition and its management.

This study revealed a diversity of beliefs among Nigerian immigrants about hypertension. Its findings show that although there may be many similarities across sub-Saharan African migrants’ beliefs and values about illnesses such as diabetes and hypertension [15, 46, 48], there may be subtle differences not previously identified in the literature. It is therefore important to explore beliefs of migrants from individual countries of origin and if possible specific in-country ethnic groups.

This study showed that Nigerian immigrants may hold multiple beliefs about the meaning of hypertension at the same time including ethnic beliefs, biomedical understanding and the idea of “hyper-tension” identified in other studies of beliefs about hypertension [42, 45, 46]. Though other studies have identified different understandings of hypertension [42, 45, 46] they have not previously reported participants holding these simultaneously.

Raised blood volume was one of the popular ethnic understandings of the meaning of the term hypertension. This is consistent with findings from the study by Taylor et al [45] conducted among diagnosed hypertensive patients living in Nigeria. However, some other meanings elicited in this study such as “burning or boiling blood”, “resistance to blood flow”, and “rising blood or blood that shoots up” have not been reported in the literature. Although, there was no clear indication of a relationship between these beliefs and perceived cause or treatment of hypertension these ideas may need further exploration in larger samples.

Moreover, although the majority of participants in this study were highly educated and had not been diagnosed with hypertension, many did not express the understanding that hypertension meant high blood pressure as measured by an instrument. This may indicate that biomedical knowledge about hypertension may be lacking among highly educated Nigerian immigrants. The belief that hypertension is a separate construct from high blood pressure was only elicited in 2 participants who were not known to be hypertensive in this study, which contrasts with the study by Taylor et al. [45] where the majority of the less well educated diagnosed hypertensive patients expressed this idea.

Overall, participants demonstrated ideas about the causes of hypertension that were similar to diagnosed hypertensive patients living in Nigeria in the study by Taylor et al [45]. Most were certain that life style such as diet, alcohol, exercise could cause hypertension or impact on the outcome of treatment of hypertension. Most thought that stress was a major cause of hypertension. Thinking too much and not having enough rest was believed to be the cause of high stress levels and by extension hypertension. These beliefs have also been reported in previous studies on Nigerians [50–55].

Interestingly, although the concept of heredity as a factor for developing hypertension was elicited in this study and other studies [39, 41, 43, 44], in our study some participants expressed the idea that hypertension was caused by inheriting poor stress management technique from one’s parents. Although this idea is congruent with the widely held belief that stress is a cause of hypertension, this finding has not been reported in other studies. This needs further exploration as to how common this belief is in the general population and understanding of what constitutes poor stress management technique.

Unusual Ideas about effects of hypertension such as hypertension causing mental health problems was elicited in this sample. This concept has not been demonstrated in other studies conducted on Nigerians [45, 50–55].

However, some participants believed that having no symptoms means not being hypertensive. Other studies conducted on sub-Saharan African migrants including Nigerians and host western populations have reported similar beliefs [41, 43, 46]. This finding is quite significant as this may impact on adherence to prescribed medications.

Unlike in the Taylor et al Nigerian hypertensive study [45], where there were gender specific differences in the area of effects of hypertension there were no differences in the perception of effects of hypertension across gender in this sample.

Although, this sample was made up predominantly of participants who were not known to be hypertensive, perceptions about treatment were similar to that expressed in the study by Taylor et al [45] and findings from other quantitative studies conducted on Nigerians [50–55].

Findings about acculturation and migration experience associated with hypertension were absent in this present study compared with other studies on migrants [14]. For example despite the fact that the concept of stress was elicited in this sample, issues related to acculturation stress like living in poor settlement areas, weather change and feelings of isolation were absent. This is probably because the majority of the participants were highly educated migrants with high income jobs. While it is not suggested that this class of individuals are not influenced by acculturation related stress, it may not always explain minority groups’ health behaviour patterns overtime [2].

### Strengths and limitations

An important strength of this study is that it explored beliefs about hypertension among a fairly wide range of ethnic groups in a Nigerian community in the United Kingdom.

However, because most of the participants in this study were not known to be hypertensives, it is possible that beliefs of those who are already known hypertensives may not have been captured. However, in most instances there was not much difference between ideas expressed by the 2 participants who were known to be hypertensive and other participants in this study.

In the same vein, some factors such as the high educational level and the young age of most of the participants may have influenced beliefs presented in this study. Even though studies on beliefs about hypertension have not demonstrated marked differences in beliefs based on age and educational background, it is suggested that further research should be done to capture the beliefs of older and the less well educated. However, even though the beliefs of older and less well educated Nigerian immigrants have not been explored, the beliefs of this age group and social class are equally important in understanding the breadth of beliefs among Nigerian immigrants.

The researcher being a member of one of the ethnic groups could have influenced the interpretation of the data due to their shared understanding of the culture. For example, this may have helped in understanding the cultural context of beliefs but could have also resulted in under exploration of some beliefs identified during the interviews and data analysis. In order to obviate this bias, another author (RM) analysed the data independently then results were compared and final thematic framework was agreed upon by all authors. In addition member checking was done.

The authors believe that data saturation was achieved as no new themes were identified in the later interviews. However, the study could have been strengthened by triangulation using another method of data collection.

### Implications

This study identified important beliefs among Nigerian immigrants about hypertension that contribute to our understanding of the management of hypertension in this group and suggests the need for further research to determine whether these beliefs are representative of this group.

In particular one important finding is that within the same ethnic group there may be different beliefs about hypertension and individuals may hold more than one belief at the same time. If this is confirmed in further studies, the implication for practice is that health care providers should endeavour to elicit individual patient’s beliefs and tailor care to suit individual needs.

Another interesting finding of this study is that while most people believed that hypertension was announced by the presence of symptoms such as palpitation and headaches some people thought that the absence of symptoms meant absence of hypertension. Studies suggest people only take actions to initiate treatment of an illness when they experience certain symptoms [56]. Hence, it may not make sense to the patient to initiate life style changes for example, when there are no apparent symptoms of a disease. Moreover, even when patients develop symptoms and are treated with drugs; as soon as symptoms are alleviated they may stop taking drugs thereby adversely affecting the outcome of treatment.

## Conclusions

Eliciting beliefs from individuals about hypertension is important because it may help to create culturally appropriate care plans and blood pressure management programs. It may also help to improve the relationship between health care providers and patients because differences between health care providers and patients’ explanatory models may be the reason for poor communication [47, 57]. Appreciation of the beliefs of individuals about hypertension could help optimise self management and treatment among Nigerians living in the UK.
